# Profiling of the Conjunctival Bacterial Microbiota Reveals the Feasibility of Utilizing a Microbiome-Based Machine Learning Model to Differentially Diagnose Microbial Keratitis and the Core Components of the Conjunctival Bacterial Interaction Network

**DOI:** 10.3389/fcimb.2022.860370

**Published:** 2022-04-26

**Authors:** Zhichao Ren, Wenfeng Li, Qing Liu, Yanling Dong, Yusen Huang

**Affiliations:** ^1^ Qingdao University Medical College, Qingdao, China; ^2^ Qingdao Eye Hospital of Shandong First Medical University, Qingdao, China; ^3^ State Key Laboratory Cultivation Base, Shandong Provincial Key Laboratory of Ophthalmology, Eye Institute of Shandong First Medical University, Qingdao, China; ^4^ Department of Medical Oncology, The Affiliated Hospital of Qingdao University, Qingdao, China

**Keywords:** microbial keratitis, ocular microbiome, machine learning, diagnosis, core microbiota, kinless hubs

## Abstract

Both healthy and diseased human ocular surfaces possess their own microbiota. If allowed, opportunistic pathogens within the ocular microbiota may cause microbial keratitis (MK). However, the nonpathogenic component of the ocular microbiota has been proven to undermine the performance of culture, the gold standard of the etiological diagnosis for MK. As the conjunctival bacterial microbiota generates unique alterations with various oculopathies, this study aimed to evaluate the feasibility of distinguishing MK using machine learning based on the characteristics of the conjunctival bacterial microbiome associated with various types of MK. This study also aimed to reveal which bacterial genera constitute the core of the interaction network of the conjunctival bacterial microbiome. Conjunctival swabs collected from the diseased eyes of MK patients and the randomly chosen normal eyes of healthy volunteers were subjected for high-throughput 16S rDNA sequencing. The relative content of each bacterial genus and the composition of bacterial gene functions in every sample were used to establish identification models with the random forest algorithm. Tenfold cross validation was adopted. Accuracy was 96.25% using the bacterial microbiota structure and 93.75% using the bacterial gene functional composition. Therefore, machine learning with the conjunctival bacterial microbiome characteristics might be used for differentiation of MKs as a noninvasive supplementary approach. In addition, this study found that *Actinobacteria*, *Lactobacillus*, *Clostridium*, *Helicobacter*, and *Sphingomonas* constitute the core of the interaction network of the conjunctival bacterial microbiome.

## 1 Introduction

Microbial keratitis (MK) is an infection of the cornea and mainly includes bacterial keratitis (BK), fungal keratitis (FK), and viral keratitis (VK). (Austin et al., 2017) MK is the fourth leading cause of blindness worldwide, and its prevalence differs in developing and developed countries, (Austin et al., 2017; Khor et al., 2018) with the estimated incidence in developed countries ranging from 6 to 40 per 100,000 and from 113 to 799 per 100,000 in developing countries. (Ung et al., 2019)

Although the specific symptoms, outcomes and prognosis depend on the pathogen species, if there is a lack of appropriate treatment, MK usually results in a series of devastating consequences, such as severe corneal inflammation, ulcers, perforations, endophthalmitis and even eye enucleation. (Prajna et al., 2013; Lobo et al., 2019) Systemic and topical antimicrobial treatment as the current recommended preoperative treatment is of vital importance. (Sharma et al., 2010) Nevertheless, if appropriate antimicrobial treatment is delayed, patients may have to receive therapeutic keratoplasty, which has a definitive role in the treatment of refractory MK. (Sharma et al., 2010) However, there is a large discrepancy between the number of donor corneas and patients waiting for keratoplasty, with a ratio of approximately 1:70. (Gain et al., 2016) Thus, as antibacterial, anti-fungal and anti-viral medications differ, accurate identification of different types of corneal infections is crucial for appropriate treatment. Microbial culture is currently considered the gold standard for the etiological diagnosis of MK. (Sharma, 2012; Ren et al., 2020) However, its positive rates are often unsatisfactory, (Wang et al., 2019; Ren et al., 2020) and atypical morphology may cause misjudgment. (Ren et al., 2020) Moreover, the nonpathogenic component of the human conjunctival and corneal microbiota can also interfere with culture positivity, which adversely affects the reliability of culture. (Ren et al., 2020)

Pathogenic bacteria, fungi and viruses induce keratitis by secreting exotoxins, releasing mycotoxins, and interacting with host cells to trigger an inflammatory cascade. (Lakhundi et al., 2017; Lobo et al., 2019) That is, different infections may inflict diverse selective pressure on ocular microbiota and result in unique alterations of conjunctival bacterial microbiota. (Danne et al., 2021; Ecklu-Mensah et al., 2022) To be specific, the host–pathogen interface is a battlefield where reactive substances and harsh microenvironment are produced by both the host and the pathogen. (Baishya and Wakeman, 2019; Li et al., 2020) One of the harsh challenges faced by invading pathogens is the limitation of essential nutrients, which are actively sequestered by the host immune response - nutritional immunity. (Baishya and Wakeman, 2019) Besides, while invading host tissues, pathogenic microbes also face stresses presented by surrounding microbes in the form of antimicrobial molecules and competition for nutrients. (Baishya and Wakeman, 2019) Thus, to establish infection, invading pathogens would continuously at war with both host cell and other microbial species within the sites of infection. The specific types of victorious invasion thus may be inferred by surrounding microbes in the battlefield. Current high-throughput 16S rRNA gene sequencing can reveal almost all bacterial genera in a conjunctival swab with a nearly one hundred percent success rate for examined samples. (Ozkan and Willcox, 2019; Ren et al., 2021) Therefore, the conjunctival bacterial microbiome might be used to differentially diagnose MK.

Machine learning is an umbrella term for algorithms that attempt to extract hidden patterns from vast amounts of known data and use them to predict or classify new data. With advances in computer science, machine learning has allowed for precision clinical diagnosis, such as recognizing clinical images and screening biomarkers. (Xiang et al., 2020) Therefore, this study aimed to evaluate the feasibility of combining the conjunctival bacterial microbiome with machine learning to differentially diagnose MK.

The Human Microbiome Project was initiated by the National Institutes of Health in 2007 to identify the microorganisms that reside normally on the healthy human body and ultimately characterize changes associated with disease states. (Consortium IHRN, 2019) Using high-throughput sequencing technology, our group has defined the normal core conjunctival microbiota, (Huang et al., 2016; Wang et al., 2019) reported alteration of the conjunctival microbiome in diverse ophthalmic diseases, (Dong et al., 2019; Ge et al., 2019) revealed the influence of different eyedrops on the conjunctival microbiome, (Fan et al., 2020) and deduced a possible factor rendering an individual vulnerable to BK from the perspective of the conjunctival bacterial microbiota. (Ren et al., 2021) The present study additionally aimed to reveal the alterations in the conjunctival bacterial microbiome in MK patients and to deduce the bacterial interaction network on the conjunctiva and significant bacterial genera within the network according to alteration of the conjunctival bacterial microbiome related to different types of infection.

## 2 Materials and Methods

### 2.1 Ethics Approval

This research was approved by the Ethics Committee of Qingdao Eye Hospital (2019-16, 11/Dec/2019) and registered on the Chinese Clinical Trial Registry (ChiCTR2000031875, 14/Apr/2020). All procedures complied with the tenets of the Declaration of Helsinki. Altogether, 69 healthy subjects and 80 patients with different MK were included in this study. Informed consent was obtained from all participants. Detailed information about the subjects is shown in **Table S1**.

### 2.2 Preliminary Screening Criteria for Microbial Keratitis

Bacterial keratitis (BK) was preliminarily considered if patients met at least two of the following criteria: (Bourcier et al., 2003; Ruiz Caro et al., 2017; Chidambaram et al., 2018) ① a history of corneal abrasion during contact lens wearing; ② diabetes mellitus; ③ immunosuppressive treatment; ④ sudden onset of ulceration; and ⑤ an ulcer with a well-defined border on slit-lamp microscopy (except for wreath-like infiltrates in *Nocardia* keratitis) (**Figure S1A**).

Patients were suspected of having fungal keratitis (FK) if they conformed to two or more of the following conditions: (Bharathi et al., 2003; Jain et al., 2007; Alkatan and Al-Essa, 2019; Ren et al., 2020) ① a history of eye trauma related to plants or soil; ② chronic systemic or local use of antibiotics or corticosteroids; ③ corneal ulceration presenting with a dry appearance; ④ elevated lesions; ⑤ satellite lesions or pseudopods; and ⑥ presence of dense hypopyon or endothelial plaques on slit-lamp microscopy (**Figure S1B**).

Cases with any two or more of the following conditions were regarded as suspected viral keratitis (VK): (Kim et al., 2018; Valerio and Lin, 2019; Erdem et al., 2020), ① repeated recurrences; ② constitutional symptoms of viral infection, such as influenza symptoms and fever; ③linear and branching corneal dendrites with terminal bulbs; ④irregular geographic ulcer or iris atrophy on slit-lamp microscopy (**Figure S1C**); ⑤ feeble response to empiric antibacterial, anti-fungal, and anti-parasitic treatment; ⑥ abundant blisters under the corneal epithelium or dendritic cells on *in vivo* confocal microscopy (IVCM); and ⑦ characteristics of viral infection in tissue sections of the corneal lesions stained with hematoxylin-eosin.

### 2.3 Diagnostic Methods and Criteria

#### 2.3.1 *In vivo* Confocal Microscopy

A confocal microscope HRT3 (Heidelberg Engineering, Heidelberg, Germany) was used to detect suspected FK cases at 800x magnification. If any fungal hyphae or spores (**Figure S1E**) were visible on the cornea, the result was positive for FK.

#### 2.3.2 Corneal Scraping Smear

Corneal scrapings were obtained using an ophthalmic microsurgical knife (Cat. No. MR-G137A, Suzhou Mingren Medical Equipment Co., Suzhou, China) under a microscope in an operating room and smeared onto a sterile glass slide. Gram staining was performed for bacteria (**Figure S1D**) and 10% potassium hydroxide (KOH) for fungi (**Figure S1H**) by microscopy at 1000x magnification and 400x magnification. If any bacterial or fungal hyphae or spores were observed, the result was reported as positive for BK or FK. (Ren et al., 2020)

#### 2.3.3 Corneal Ecraping Culture

Corneal scrapings were quad plate-streaked on blood agar medium (Cat. No. 16, Autobio, Zhengzhou, China) for bacterial culture and Sabouraud dextrose agar medium (Cat. No. A0697077, Baibo Biotechnology, Jinan, China) for fungal culture. The bacterial and fungal cultures were incubated at 37°C and 25°C, respectively, with daily observation. (Mascarenhas et al., 2014; Austin et al., 2017; Ren et al., 2020) Once macroscopic colonies (As shown in **Figures S1G, H**) appeared on the agar medium, after colonies being stained with Gram stain for bacteria and with lactophenol cotton blue stain for fungi, the type of infection was judged by two experienced technicians according to the growth rate, color, margin and surface and changes in colonies during the period of culture, as well as the staining characteristics under a microscope (**Figures S1G, H**). (Ren et al., 2020) If corneal scraping smear and culture were both positive, the organisms on media having the same morphology to the smear would be deemed as the causative pathogen and recorded in **Table S1**. If no colonies formed within 14 days, the culture was reported to be negative.

#### 2.3.4 Viral Quantitative Real-Time PCR

For intractable VK cases, corneal scrapings were sent for qPCR to confirm a diagnosis. qPCR was performed by KingMed Diagnostics Group Co., Ltd. (Guangzhou, China). Total RNA was extracted using the EZ1 Virus Mini kit v2.0 on the EZ1 automated extraction platform (955134, Qiagen, Germany). Real-time qPCR was carried out using an Applied Biosystems 7500 Real Time PCR System (Applied Biosystems, Foster, USA), using the following parameters: an initial denaturation step at 95°C for 10 minute, followed by 50 cycles of amplification (95°C for 5 s; 62°C for 30 s; and 66°C for 15 s).

Herpes zoster virus primers: 5’-GATTACAGGCGAGCCCATTAGA-3’ and 5’-CGTATCGGGACTTCAACCAGAA-3’. (Schmid et al., 2021) Herpes simplex virus primers: 5’-GGGGTGATCGGCGAGTAYTG-3’ and 5’-ATCTGCTGGCCGTCGTARATG-3’. (Tan et al., 2013) Vaccinia virus primers: 5’-AAACACACACTGAGAAACAGCATAAA-3’ and 5’-ACTATCGGCGAATGATCTGATTA-3’. (Huttunen and Mercer, 2019) Adenovirus primers: 5’-GCCACGGTGGGGTTTCTAAACTT-3’ and 5’-GCCCCAGTGGTCTTACATGCACATC-3’. (Heim et al., 2003).

### 2.4 Inclusion and Exclusion Criteria

During the recruiting, based on diagnostic and exclusion criteria, all definitely diagnosed patients with MK and healthy volunteers were included in this study. Subjects with any recent diagnosis and treatment at other eye care institutions, recent usage of any eyedrops (such as artificial tears and low-concentration atropine), any systemic drug therapy, concurrent eye diseases, or any previous oculopathy or ophthalmic/corneal refractive surgery were excluded.

### 2.5 Conjunctival Swab Collection

For patients with MK, the conjunctival cotton swab from all diseased eyes were collected and the fellow eyes were untreated. Subjects lay supine in a disinfected operating room (conforming to GB50333-2013-I standard of China), with the facial area except the examined eye covered with a sterile surgical drape after eyelid disinfection with iodophor. A drop of oxybuprocaine hydrochloride eye drops was used for anesthesia before sampling. (Ren et al., 2020) The superior palpebral conjunctiva, superior bulbar conjunctiva, inferior bulbar conjunctiva, inferior fornical conjunctiva, and inferior palpebral conjunctiva of the examined eye were rubbed lightly in sequence using one sterile cotton swab (Katzka et al., 2021) (from the supply department of Qingdao Eye Hospital of Shandong First Medical University according to T/CSBME013-2019 standard of China) soaked with oxybuprocaine hydrochloride eye drops. (Ren et al., 2020) The meibomian orifices, eyelashes, and cornea were avoided.

For control group, a randomly chosen single eye of each healthy subject was treated in the same way. Besides, three sterile swabs were exposed to laminar flow for 3 minutes; after intraday samples were obtained, the remaining oxybuprocaine hydrochloride eye drops were sent for contamination analysis by high-throughput 16S rRNA gene sequencing. If negative results were generated, the collected swabs were used for DNA extraction and sequencing.

### 2.6 High-Throughput 16S rRNA Gene Sequencing

#### 2.6.1 DNA Extraction

Total genomic DNA was extracted from conjunctival swabs using DNA Extraction Kit (Cat. No. D3096-100T, Omega Bio-Tek, Norcross, GA, USA). All DNA samples were delivered to OE Biotech (Qingdao, China) for the procedures described below.

#### 2.6.2 DNA Amplification

In total, 50 ng of DNA without degradation or with slight degradation (verified by OD260/280 ranges within 1.6-1.8, and 0.6% agarose gel electrophoresis with 120 V constant voltage for 15 minutes) was diluted to 1 ng/μl as a template and used for PCR amplification (Cat. No. 580BR10905, Bio–Rad, Richmond, CA, USA), with primers and Takara Ex Taq (Cat. No. RR001Q, Takara, Japan). V3-V4 variable regions of the 16S rRNA gene were amplified using universal primers 343F (5’-TACGGRAGGCAGCAG-3’) and 798R (5’- AGGGTATCTAATCCT-3’).

#### 2.6.3 Library Construction

Amplicon quality was refined with AMPure XP beads (Agencourt, Brea, CA, USA), and the refined DNA was enlarged for another round using primers 343F and 798R again. Ultimately, the amplicon was quantified using the Qubit dsDNA assay kit (Cat. No. Q32852, Life Technologies, Carlsbad, CA, USA) to obtain the final amplicon after purification with AMPure XP beads again. Equal amounts of refined amplicon were sent for subsequent high-throughput sequencing using Illumina MiSeq Pe300 (Illumina, San Diego, CA, USA).

#### 2.6.4 Bioinformatic Analysis

Raw sequencing data were stored in FASTQ format and uploaded into NCBI BioProject database (accession no: PRJNA695410). Sequencing results were subjected to primer sequence removal using QIIME software (version 1.8.0). (Caporaso et al., 2010) Paired-end reads were analyzed using Trimmomatic software (version 0.53) (Bolger et al., 2014) to identify and remove ambiguous bases (N). Trimmomatic software also removed inferior-quality sequences with an average quality score below 20 through the sliding window trimming approach. Then, paired-end reads were assembled using FLASH software (version 1.2.11). (Reyon et al., 2012) The parameters of assembly were 10 bp of minimal overlapping, 200 bp of maximum overlapping and 20% of maximum mismatch rate. Sequences were further denoised as follows: reads with ambiguous, homologous sequences or below 200 bp were abandoned. Using QIIME software, reads with 75% of bases above Q20 were retained, and reads with chimeras were detected and removed. Then, clean reads were clustered to form operational taxonomic units (OTUs) with 97% similarity using the Vsearch software (version 2.4.2, available at https://github.com/torognes/vsearch). All representative reads of every OTU were chosen using the QIIME package and annotated and blast-searched against the Silva database (version 123) and Greengens database using the RDP classifier with a confidence threshold value of 70%. (Wang et al., 2007) More details about the software parameters are shown in **Table S2**.

PICRUSt2 software (available at https://github.com/picrust/picrust2) were used to analyze the functional composition of bacterial genes, and differences for different samples (Kruskal–Wallis; the threshold of P value was 0.05) and groups (T test; the threshold of P value was 0.05) were determined based on the KEGG database. (Langille et al., 2013) LEfSe (linear discriminant analysis coupled with effect size measurements), a high-dimensional class analysis to determine the microorganism most likely to account for differences between groups (biomarkers). (Dong et al., 2019) Using the statistical software R (corrplot package, available at https://github.com/taiyun/corrplot), a predicted interaction network of the top 30 bacterial genera was established based on the Spearman correlation coefficient. Among the interaction network, bacterial genera with |SpearmanCoef| > 0.8 and P < 0.01 are highlighted.

All procedures from DNA amplification to bioinformatic analysis were performed by technicians at OE Biotech (Qingdao, China). More details about these procedures are available at https://www.qdoebiotech.com under contract number (OE2018H2240V-2&OE2018H2239V).

### 2.7 Establishment of Identification Models

The relative content of each bacterial genus and the composition of bacterial gene functions in a sample were used to establish identification models based on the random forest algorithm, which can combine numerous randomized decision trees and aggregate all predictions by averaging. (Biau and Scornet, 2016) The random forest algorithm has excellent performance when variables are much larger than the number of observations. (Biau and Scornet, 2016) Moreover, it is versatile enough to be applied to large-scale problems. (Biau and Scornet, 2016)

Tenfold cross-validation was adopted to reduce the risk of overfitting and bias. (Lin et al., 2020) Briefly, all samples were randomly divided into 10 subsamples with stratified sampling. One single subsample served as the testing data; the remaining 9 subsamples were retained as the training data to construct an identification model. The process was repeated 10 times (folds) and generated an average accuracy to evaluate the final identification model, with each subsamples used exactly once as the testing data.

### 2.8 Performance Evaluation of the Model

A confusion matrix, which helps to quickly visualize the proportion of categories that are misclassified into other categories, and a receiver operating characteristic (ROC) curve were used to intuitively demonstrate the performance of the identification models. For a ROC curve, the closer the area under the ROC curve (AUC) is to 1, the better the authenticity of the diagnostic model is.

The precision, recall, and F1-score were employed for statistical analyses of each class. Precision is the ratio of the number of correctly classified samples to the total number of classified samples [True Positive/(True Positive + False Positive)]. Recall is the ratio of the number of correctly identified samples to the number of samples that should have been identified [True Positive/(True Positive + False Negative)]. The F1-score is generally used to measure the accuracy of unbalanced data, and it takes into account both the accuracy and recall of classification models. That is, the F1-score can be regarded as a weighted average of model accuracy and recall rate, with a maximum value of 1 and a minimum value of 0.

Macro-F1, micro-F1, weight-F1, sum recall and sum accuracy were used to statistically describe the performance of the established model. Macro-F1 is the average value of the F1-score in all classes. Micro-F1 is suitable for the condition of multicategory imbalance, in which data are extremely imbalanced and will affect the results. Weighted-F1 is the average of each F1-score multiplied by the proportion of the corresponding class. Sum accuracy indicates the ratio of the number of samples correctly classified by the model to the total number of samples.

## 3 Results

### 3.1 Overview of the Study

**Figure 1:**
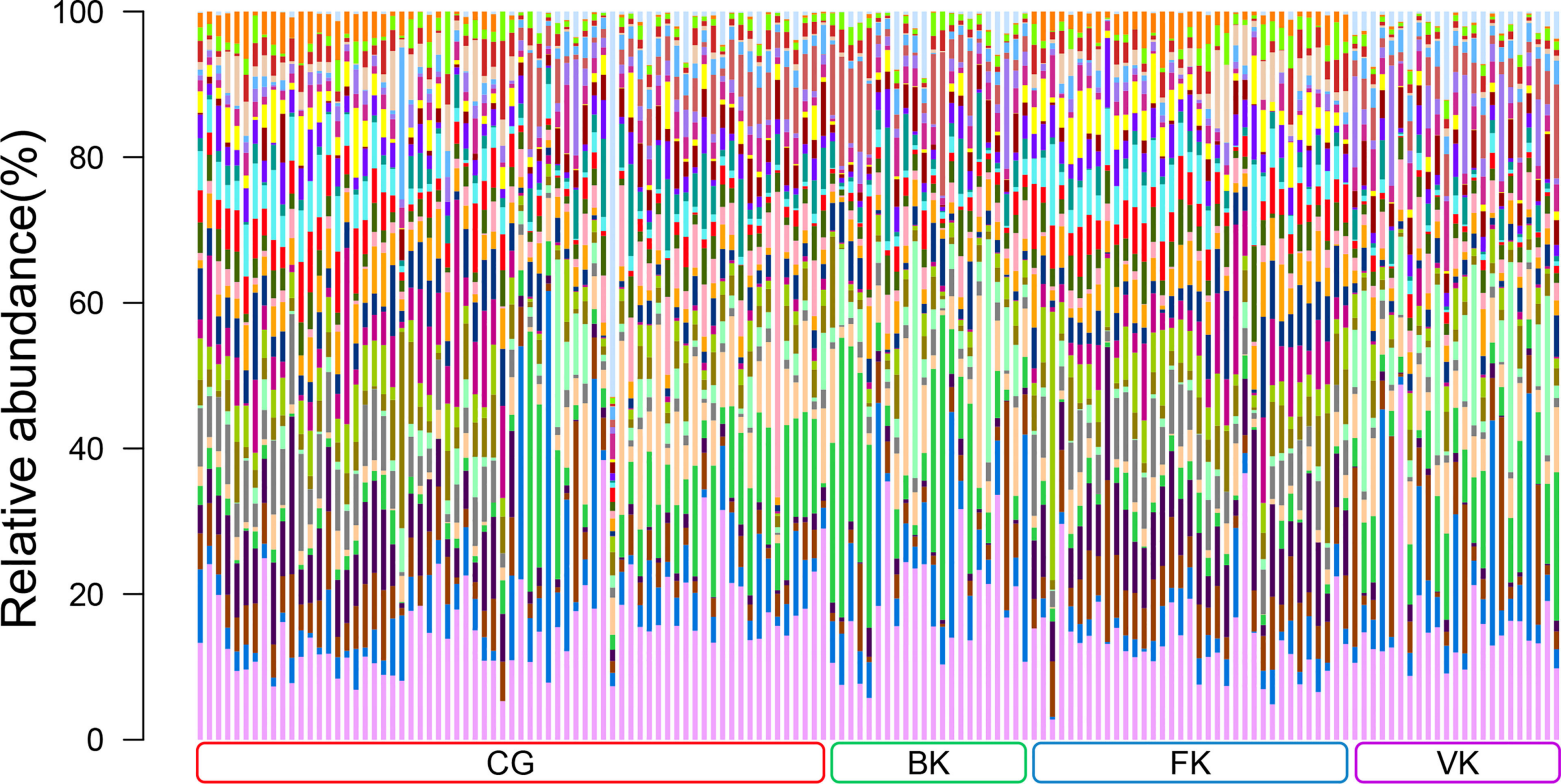
Relative abundances of the top 30 bacterial genera in the conjunctival microbiota. Each bar represents a sample of conjunctival microbiota, various color portions represent different genera, and the length of a colored portion represents the content of a genus. Among the 149 samples, 69 were from healthy eyes (CG), 22 were from bacterial keratitis (BK), 35 were from fungal keratitis (FK), and 23 were from viral keratitis (VK). Different samples possess diverse ocular surface microbiotas.

According to the results of traditional diagnostic approaches to MK (representative images are presented in **Figure S1**), a total of 149 conjunctival swabs were obtained for further study, including 69 from healthy eyes, 22 from eyes with BK, 35 from eyes with FK, and 23 from eyes with VK. The detailed information for each subject is shown in **Table S1**. The total number of OTUs in this study was 28,659, with the number of OTUs in each sample ranging from 232 to 2873. The content and representative sequence of each OTU in each sample are shown in **Supplementary Data**. The relative bacterial compositions of each sample are presented in **Figure 1**.

### 3.2 The Conjunctival Bacterial Microbiome Presented Unique Characteristics for Different Types of MK

**Figure 2:**
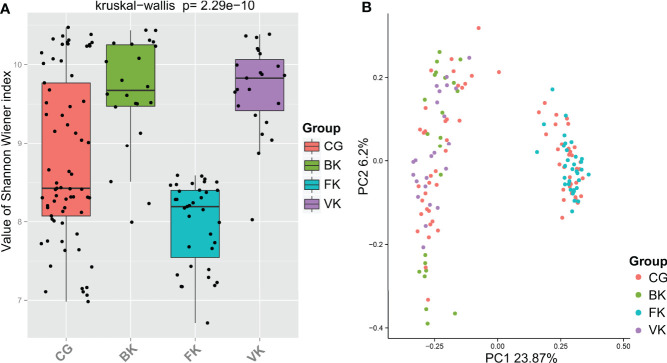
For each specific type of microbial keratitis, the ocular bacterial microbiota presents a unique community structure. **(A)** The Shannon–Wiener index reflects the species richness and evenness in a sample. **(B)** Based on dimensionality reduction, PCoA plots reveal similarities in community structure among microbiotas related to various types of microbial keratitis. (CG, control group - healthy eyes; BK, bacterial keratitis; FK, fungal keratitis; VK, viral keratitis).

The Shannon–Wiener index, an estimator of species richness and evenness that delineates within-community characteristics, differed significantly (P=2.29e−10, Kruskal–Wallis) among the four groups (**Figure 2A**). Based on dimensionality reduction, the PCoA plot (Bray Curtis algorithm; P=0.001, R^2 =^ 1, F. Model=8.269, total SumsOfSqs=43.842, and Df=148), an approach to exhibit between-community characteristics, revealed samples from the eyes with BK, FK and VK gathered in a specific region (**Figure 2B**). **Figure 2B** also illustratively suggested the conjunctival bacterial microbiota in the three types of MK presented minor intergroup differences but major between-group differences.

Furthermore, based on the Kruskal–Wallis algorithm, we used PICRUSt2 with KEGG database results to screen differentially expressed genes in different samples and to generate a heatmap (**Figure S2**). The weighted nearest sequenced taxon index (NSTI) of each sample is given in **Table S3**, with a mean value of 0.13 ± 0.02. Although the predictive accuracy may not be ideal, the heatmap clustering suggests that the three groups present a tendency of gathering related to grouping. As a result, the conjunctival bacterial microbiota may be used to distinguish MKs.

### 3.3 The Conjunctival Bacterial Microbiome can be Used to Distinguish MK With the Aid of Machine Learning

**Figure 3:**
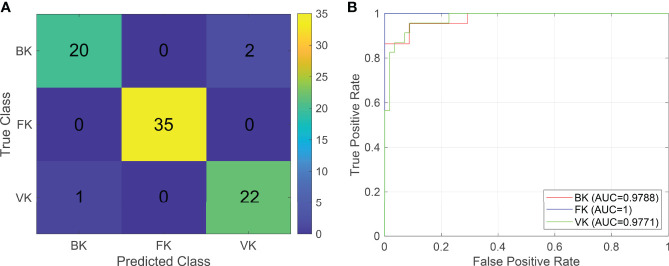
The classifier based on the relative composition of conjunctival bacterial microbiota can effectively distinguish various types of microbial keratitis. For the confusion matrix **(A)**, a number in a grid denotes how many actual cases from the ordinate are judged as the abscissa. For the ROC curve **(B)**, the AUC reflects the performance of the established classifier for distinguishing various types of microbial keratitis. (CG, control group - healthy eyes; BK, bacterial keratitis; FK, fungal keratitis; VK, viral keratitis; AUC, area under the ROC curve).

Two machine learning models were established in this study based on the relative composition of conjunctival bacterial microbiota (**Supplementary Data – Model 1**) and the composition of bacterial gene functions (**Supplementary Data – Model 2**). The sum accuracy of the former model was 96.25% (**Figure 3**); that of the latter model was 93.75% (**Figure S3**). The evaluation indexes of the machine learning model are provided in **Table 1**. As these shown, these two models can both effectively distinguish different types of MK.

**Table 1:** Performance evaluation of the two established models.

Evaluation indicators	Microbiota composition			Gene functional composition		
	BK	FK	VK	BK	FK	VK
AUC	0.9788	1	0.9771	0.9702	1	0.9741
F1	0.9302	1	0.9362	0.9091	0.9859	0.8889
Precise	0.9524	1	0.9167	0.9091	0.9722	0.9091
Recall	0.9091	1	0.9565	0.9091	1	0.8696
Macro F1	0.9558			0.9282		
Micro F1	0.9625			0.9375		
Weight F1	0.3209			0.3124		
Sum recall	0.9625			0.9375		
Sum accuracy	0.9625			0.9375		

BK, bacterial keratitis; FK, fungal keratitis; VK, viral keratitis.

The NSTI quantifies how closely a genome database represents the community of OTUs. (Douglas et al., 2018) As PICRUSt’s inference accuracy has negative relevance with regard to NSTI values, the weighted NSTI value can be applied to determine the confidence in functional inferences. (Douglas et al., 2018) The accuracy of PICRUSt decreases rapidly with increasing weighted NSTI within an NSTI below 0.2, specifically, R^2^ drops below 0.5 for genomes with weighted NSTIs above 30%. (Louca et al., 2018) According to the estimation from Stilianos Louca et al., (Louca et al., 2018) the accuracy of PICRUSt is approximately 70% when the weighted NSTI is 0.13 ± 0.02. Therefore, the relatively low accuracy of the model based on the composition of bacterial gene functions, to some extent, can be attributed to the substantial additional errors imported during the procedure of PICRUSt.

### 3.4 *Actinobacteria*, *Lactobacillus*, *Clostridium*, *Helicobacter*, and *Sphingomonas* May Constitute the Core Interaction Network of the Human Conjunctival Bacterial Microbiome

**Figure 4:**
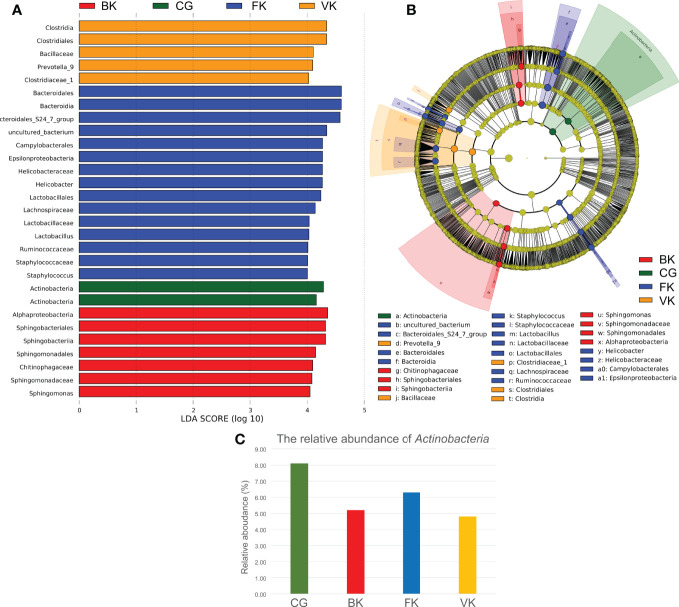
LEfSe indicates that *Actinobacteria* may be valuable and meaningful for the ocular microbiota. **(A)** When the score of a taxon is >4.0 with P < 0.01, biomarkers of different taxa for each group are listed in the histogram. **(B)** The relationship among these taxa is exhibited using a concentric circle figure. The circles, from inside to outside, show the phylum, class, order, family, and genus. The colored spots indicate biomarkers of each group. **(C)** According to the above pictures, the relative content of *Actinobacteria* decreases in microbial keratitis. (CG, control group - healthy eyes; BK, bacterial keratitis; FK, fungal keratitis; VK, viral keratitis).

According to LEfSe (**Figure 4**), *Actinobacteria* was the biomarker (LDA score >4) in the control group. Alphaproteobacteria, Sphingobacteriales, Sphingobacteriia, Sphingomonadales, Chitinophagaceae, Sphingomonadaceae, and *Sphingomonas* were biomarkers in the BK group. Bacteroidales, *Bacteroidia*, Campylobacterales, Epsilonproteobacteria, Helicobacteraceae, *Helicobacter*, Lactobacillales, Lachnospiraceae, Lactobacillaceae, *Lactobacillus*, Ruminococcaceae, Staphylococcaceae, and *Staphylococcus* were biomarkers in the FK group, and *Clostridia*, Clostridiales, Bacillaceae, *Prevotella*, and Clostridiaceae were biomarkers in the VK group. Interestingly, the average relative content of *Actinobacteria* was reduced by 35.84%, 22.41% and 40.76%, respectively, when the above three types of MK occurred.

**Figure 5:**
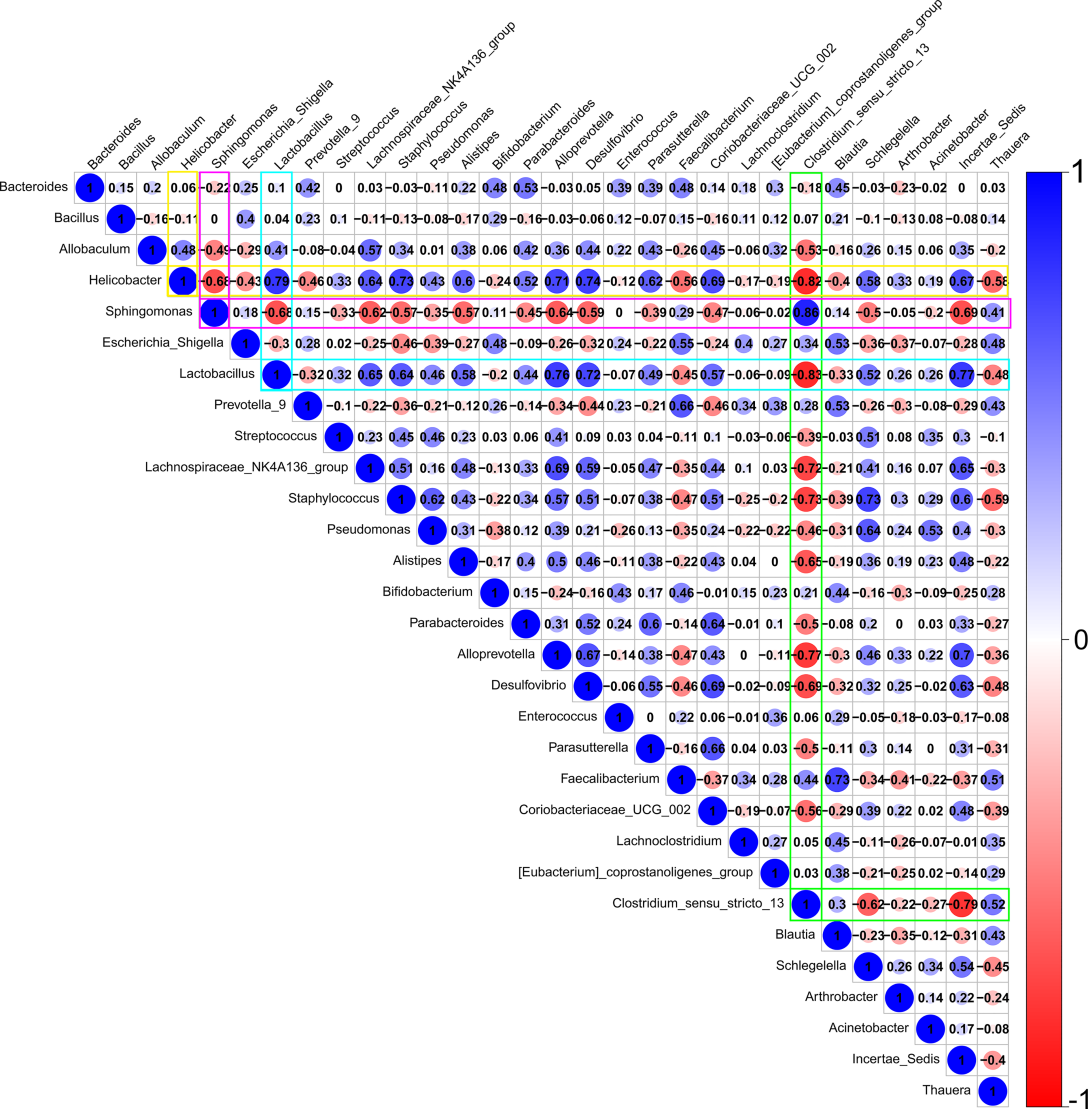
A predicted interaction network of the top 30 bacterial genera was established based on Spearman correlation coefficients. Red represents a negative correlation, and blue represents a positive correlation. Darker color, larger circle, and larger number represent a stronger correlation. *Lactobacillus*, *Clostridium*, *Helicobacter*, and *Sphingomonas* are speculated to have significant relatedness (|SpearmanCoef|> 0.8 and p <0.01) in the interaction network of the conjunctival bacterial microbiome. *Lactobacillus* may have an antagonistic effect on *Clostridium*. *Helicobacter* may have a synergistic effect on *Lactobacillus*.

**Figure 5** reveals a predicted intricate interaction network of the top 30 bacterial genera based on the Spearman correlation coefficient. *Lactobacillus*, *Clostridium*, *Helicobacter*, and *Sphingomonas* were speculated to have significant relatedness (|SpearmanCoef|> 0.8 and P <0.01) in the interaction network of the conjunctival bacterial microbiome. *Lactobacillus* exhibited a strong negative correlation with *Clostridium*, whereas *Helicobacter* might exert a synergistic effect on *Lactobacillus*.

## 4 Discussion

### 4.1 The Conjunctival Bacterial Microbiome May be a Noninvasive Supplementary Approach to Distinguish MK

The current clinical application of high-throughput 16S rRNA gene sequencing generally aims to verify bacterial infection and to reveal causative bacteria in airtight tissue, such as articular cavity and cardiac valve tissue. (Vondracek et al., 2011; Larsen et al., 2018) However, high-throughput 16S rRNA gene analysis cannot verify bacterial infection in tissues with a microbiota. High-throughput 16S rRNA gene sequencing of conjunctival swabs can achieve a nearly one hundred percent success rate, while the positivity rate of bacterial culture for the ocular surface is only approximately 60% and that of fungal culture ranges from 3% to 65%. (Huang et al., 2016; Wang et al., 2019; Ren et al., 2020) In addition, conjunctival and corneal microbiome have very similar microbial taxonomic profiles with different proportion. (Matysiak et al., 2021) Besides, previous studies have confirmed that different types of pathogens can invoke various ocular pathophysiological responses. (Suzuki et al., 2010; Lobo et al., 2019) Our present work is thus aimed to extend the clinical application of high-throughput 16S rRNA gene sequencing, especially for the tissue diagnosis associated with a microbiota.

In this study, BK, FK, and VK impose diverse selective pressures on the conjunctival bacterial microbiome, and specific bacteria may survive in such a specific circumstance. Besides, the conjunctival bacterial microbiota in three types of MK presented minor intergroup differences and major between-group differences, though many other factors are likely involved, including age, (Zhou et al., 2014; Wen et al., 2017) gender, (Wen et al., 2017) season, (Zhou et al., 2014) and contact lens wear (Shin et al., 2016). That is, the selective pressures of different types of corneal infection on the conjunctival bacterial microbiome appear to be more influential than the aforementioned individual factors, which also suggests microbiome-based machine learning possesses commendable robustness (Lesne, 2008) for differentially diagnosing MK.

A previous study has shown that well-covered human samples (such as gut samples) possess the lowest NSTI (mean NSTI = 0.03 ± 0.02) but that the NSTI of soils is 0.17 ± 0.02 and that of other mammal samples is 0.14 ± 0.06. (Langille et al., 2013) That is, PICRUSt does not offer outstanding performance for conjunctival samples, as it does for gut samples. Undoubtedly, shotgun metagenomic sequencing or metagenomic whole-genome sequencing can theoretically generate a more accurate deduction of bacterial gene functions. Nevertheless, host DNA contamination can overwhelm the low biomass of microbial signals and decrease sensitivity for microbial detection, (Heravi et al., 2020) especially for infected ocular samples, which usually contain abundant host cells. As a result, though the accuracy of the identification models using the bacterial gene functional composition inferred by PICRUSt are conservative, the identification models using the structure of the bacterial microbiota may have a broader practicability in clinical application.

In 2014, Yoshio Nakano et al. (Nakano et al., 2014) reported the first combined clinical application of the microbiota and machine learning, presenting a model to classify oral malodor based on the microbiota in saliva samples. Two years later, Kentaro Iwasawa et al. (Iwasawa et al., 2018) found that dysbiosis of the salivary microbiota can be used to diagnose pediatric-onset primary sclerosing cholangitis. Since 2020, previous studies have combined the microbiota and machine learning for the disease diagnosis, classifying and prognosis forecasting. (Willis et al., 2020) Regardless, the present study adds to our knowledge by first using this approach to distinguish various types of infections in tissues that have a microbiota.

With the development of nanopore sequencing, on-site sequencing has gradually been implemented in clinical applications. For example. MinION is a portable (only weighs 90 g and can plug into any computer with a standard USB 3.0 port), real-time device for DNA and RNA sequencing. In addition, the identification model of machine learning has a very low marginal cost (an increase in the total cost caused by newly generated results). (Ting et al., 2017) Therefore, approaches that combine the microbiota and machine learning are promising with regard to accessibility and affordability.

### 4.2 *Actinobacteria*, *Lactobacillus*, *Clostridium*, *Helicobacter*, and *Sphingomonas* Appear to Constitute the Core Interaction Network of the Human Conjunctival Bacterial Microbiome

To better describe and analyze the microorganisms and key genera in an environment, researchers proposed the concept of the “core microbiome”, which means that the existence of microorganisms in this group does not depend on factors such as the environment, lifestyle or physiological differences. (Turnbaugh et al., 2007; Willcox, 2013; Huang et al., 2016) Initially, the conclusion of core microbiome was deduced mainly based on the conjunctival microbiome on healthy eyes. For example, our research team (Huang et al., 2016) identified 10 genera (*Corynebacterium*, *Pseudomonas*, *Staphylococcus*, *Acinetobacter*, *Streptococcus*, *Millisia*, *Anaerococcus*, *Finegoldia*, *Simonsiella* and *Veillonella*) constituting the conjunctival core microbiome; Dong et al. (Dong et al., 2011) found 12 genera (*Pseudomonas*, *Propionibacterium*, *Bradyrhizobium*, *Corynebacterium*, *Acinetobacter*, *Brevundimonas*, *Staphylococci*, *Aquabacterium*, *Sphingomonas*, *Streptococcus*, *Streptophyta*, and *Methylobacterium*) constituting the conjunctival core microbiome, and Ham et al. (Ham et al., 2018) found 8 (*Corynebacterium*, *Streptophyta sp*, *Bradyrhizobiaceae sp*, *Sphingomonas*, *Ralstonia*, *Neisseriaceae sp*, *Acinetobacter*, and *Pseudomonas*). However, with the development of microbiome research, the concept of the core microbiome has gradually evolved from the initial “shared members of microbial communities in different samples” to “members of microbial communities that play a key role in the local ecosystem, host-microbial interactions, microbiome function, and microbiome persistence and stability”. (Chun-Bo et al., 2019) It seems that the conjunctival core microbiome needs to be defined again to determine at least some components that can guarantee ocular surface homeostasis. (Aragona et al., 2021)

Invoking the community theory of agricultural ecosystems, microbial taxa within complex ecological networks are usually categorized by their universal roles based on their level of connectivity with other taxa. (Toju et al., 2018) Highly connected taxa (kinless hubs) within an ecological network are theoretically expected to support higher levels of ecosystem functions than less connected taxa (peripherals). (Shi et al., 2020) Kinless hubs are considered to create ecological niches, i.e., the minimum habitat and resource thresholds necessary for the survival of each microorganism, for other taxa. (Shi et al., 2020) As a result, kinless hubs are of paramount importance to maintain productivity in human-managed agricultural ecosystems such as cropland and orchard. In consideration of the limitation of the concept of “core microbiome”, this study aimed to introduce the concept of “kinless hubs” and “peripherals” into the human microbiome for the first time. Accordingly, we deduced the bacterial interaction network on the conjunctiva as well as significant bacterial genera within the network according to alteration of the conjunctival bacterial microbiome related to different types of infection. *Actinobacteria*, *Lactobacillus*, *Clostridium*, *Helicobacter*, and *Sphingomonas* were found to constitute the core interaction network of the human conjunctival bacterial microbiome and can be considered kinless hubs of the conjunctival microbiota.

*Actinobacteria*, a common bacterial genus, can synthesize numerous secondary metabolites that have antimicrobial, antiviral, antiparasitic, antioxidant, anticancer, and neurological activities. (Valliappan et al., 2014; Dalitz et al., 2017) As shown by LEfSe, compared with healthy subjects, *Actinobacteria* was significantly reduced in the conjunctival microbiota of MK eyes, and our previous study (Ren et al., 2021) and that of Shivaji et al. (Shivaji et al., 2021) also reported that *Actinobacteria* is significantly reduced in the conjunctival microbiota of the diseased eyes of BK patients. Hence, *Actinobacteria* may have a potential value on the conjunctiva, similar to the performance of *Corynebacterium mastitidis* on the mouse ocular surface, which is a validated ocular probiotic for mouse that can tune ocular immunity and protecting the eye against pathogenic infection. (St Leger et al., 2017)

Furthermore, based on Spearman correlation coefficient analysis, *Sphingomonas*, as a biomarker for BK, *Lactobacillus* and *Helicobacter*, as biomarkers for FK, and *Clostridium*, as a biomarker for VK, might have significant relationships in the interaction network of the conjunctival bacterial microbiome. According to a previous *in vitro* study, (Monteiro et al., 2019) *Lactobacillus* has antimicrobial activity against *Clostridium*, which is in line with the predicted interaction network in our series. *Helicobacter* can both cause peptic ulcer disease and alleviate Barrett’s esophagus, and the predicted interaction network showed that it may cooperate with *Lactobacillus*, which is recognized as a probiotic. (O’Callaghan and O’Toole, 2013; Malnick et al., 2014; Chen et al., 2019) Nonetheless, the network in this study has not been fully verified *in vitro* and *in vivo* and further investigation is required. A completely decoded interactional network of the ocular surface microbiome will definitely better facilitate the prevention, diagnosis, and treatment of eye diseases.

There are limitations in this study. First, the sample size was inadequate to establish a versatile identification model for actual clinical application concerning different geographic areas or human races. Second, *Acanthamoeba*, an infrequent pathogenic wate-rborne parasite of MK that accounts for less than 1%, was not identified. (Zeng et al., 2008; Cohen, 2015; Austin et al., 2017) Third, the degree of acceptance of artificial intelligence algorithms in physicians and patients remains unclear. (Xiang et al., 2020) However, with microbiome data from around the world being deposited in public repositories, an ideal program for clinical use is promising.

## 5 Conclusion

This study proposes a noninvasive approach that combines machine learning with the conjunctival bacterial microbiome to distinguish MK and may also enlighten the diagnosis for other tissues associated with a microbiota. In addition, this study revealed that *Actinobacteria*, *Lactobacillus*, *Clostridium*, *Helicobacter*, and *Sphingomonas* constitute the core interaction network of the human conjunctival bacterial microbiome and should be considered kinless hubs of the conjunctival bacterial microbiota, contributing to an in-depth understanding of the ocular surface microbiome.

## Data Availability Statement

The datasets presented in this study can be found in online repositories. The names of the repository/repositories and accession number(s) can be found in the article/**Supplementary Msaterial**.

## Ethics Statement

The studies involving human participants were reviewed and approved by Ethics Committee of Qingdao Eye Hospital. The patients/participants provided their written informed consent to participate in this study. Written informed consent was obtained from the individual(s) for the publication of any potentially identifiable images or data included in this article.

## Author Contributions

ZR contributed to the design of this research, literature search, data analysis, identification model establishment, and drafting of the manuscript. WL contributed to the design of this research and revision of the manuscript. QL contributed to the conjunctival swab collection and patient management. YD contributed to the clinical diagnosis. YH contributed to the design of this research, literature search, data analysis, and revision of the manuscript All authors contributed to the article and approved the submitted version.

## Funding

This research was supported by the National Natural Science Foundation of China (81970788, 82171027), the Key Science and Technology Innovation Project of Shandong Province (2018CXGC1205), and the Taishan Scholar Program (ts20190983).

## Conflict of Interest

All authors declare that the research was conducted in the absence of any commercial or financial relationships that could be construed as a potential conflict of interest.

## Publisher’s Note

All claims expressed in this article are solely those of the authors and do not necessarily represent those of their affiliated organizations, or those of the publisher, the editors and the reviewers. Any product that may be evaluated in this article, or claim that may be made by its manufacturer, is not guaranteed or endorsed by the publisher.
