# Supplementary material for: Profiling of the Conjunctival Bacterial Microbiota Reveals the Feasibility of Utilizing a Microbiome-Based Machine Learning Model to Differentially Diagnose Microbial Keratitis and the Core Components of the Conjunctival Bacterial Interaction Network
Source: Front Cell Infect Microbiol. 2022 Apr 26;12:860370. doi: 10.3389/fcimb.2022.860370 (PMC9086711; doi:10.3389/fcimb.2022.860370)
Supplement: Supplementary file 1 [file DataSheet_1.docx]

Supplementary Material

# Supplementary Data

Supplementary Data included two model of machine learning established in this study, as well as two corresponding data set.

# Supplementary Figures and Tables

## Supplementary Figures


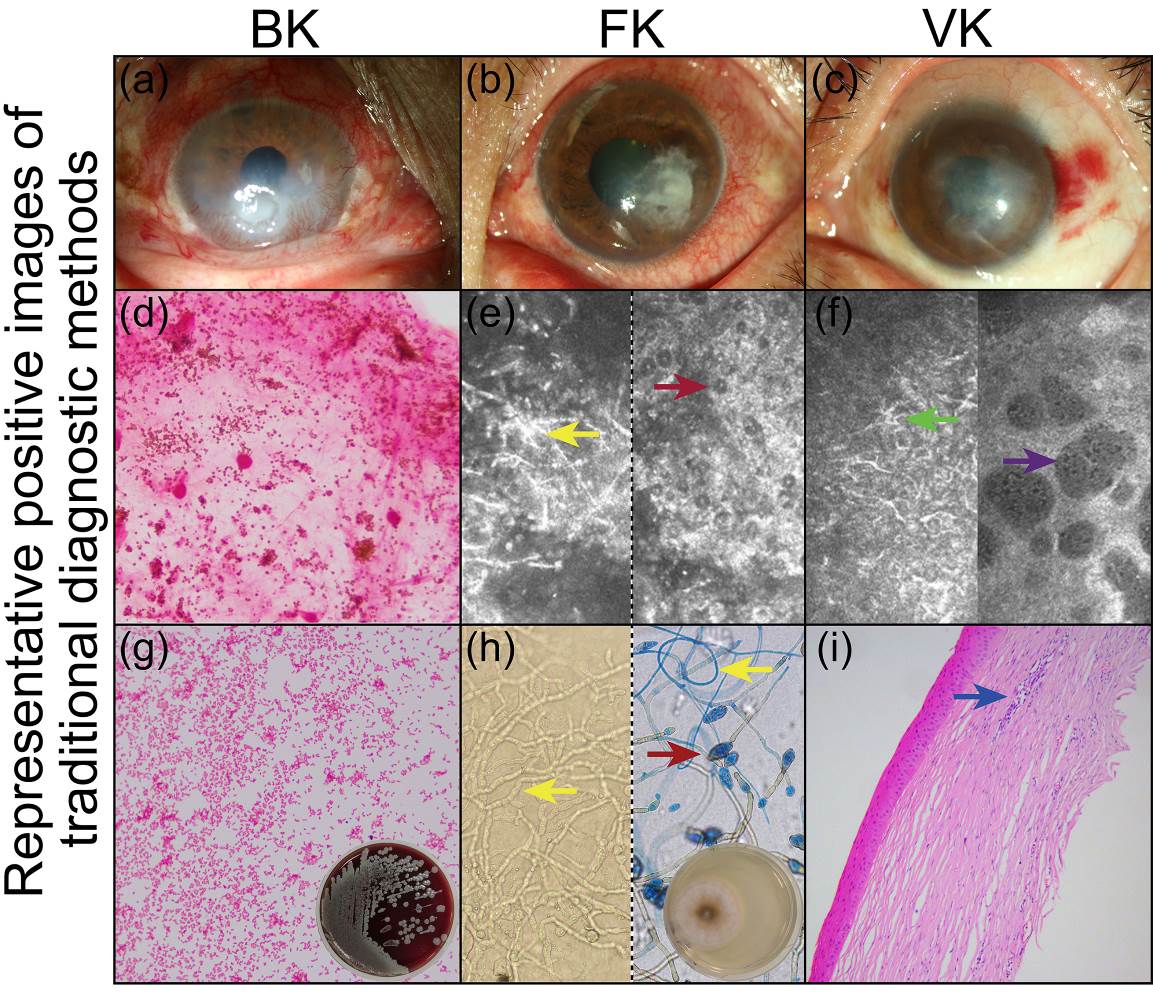


**Fig. S1.** **Representative images of positive results using traditional diagnostic methods.** **(a)** An eye with bacterial keratitis presents a sudden onset of ulceration with a well-defined border. **(b)** An eye with fungal keratitis presents an elevated corneal ulceration with dry appearance and satellite lesions and pseudopods. **(c)** An eye with viral keratitis presents an irregular geographic ulcer with linear and branching corneal dendrites with terminal bulbs. **(d)** Gram stain smear of bacteria at 1000× magnification. **(e)** Fungal hyphae and spores on IVCM at 800× magnification. The yellow arrow to the left refers to hyphae, and the red arrow to the right refers to spores. **(f)** Dendritic cells and blisters under the corneal epithelium on IVCM at 800× magnification. The green arrow to the left refers to dendritic cells, which mainly participate in the immunoreaction of viral infection. The purple arrow to the right refers to blisters under the corneal epithelium, which often occur with viral infection. **(g)** The microscopic characteristics of cultured bacterial colonies stained with Gram stain at 1000× magnification and the macroscopic characteristics of bacterial colonies in blood agar medium. **(h)** The microscopic characteristics of KOH smears and cultures of fungal colonies stained with lactophenol cotton blue stain at 400× magnification and the macroscopic characteristics of fungal colonies in Sabouraud dextrose agar medium. The yellow arrows to the left refer to hyphae, and the red arrow to the right refers to spores. **(I)** The tissue section of the corneal lesions stained with hematoxylin-eosin suggests viral infection (blue arrow). (IVCM: *in vivo* confocal microscopy; KOH: potassium hydroxide).


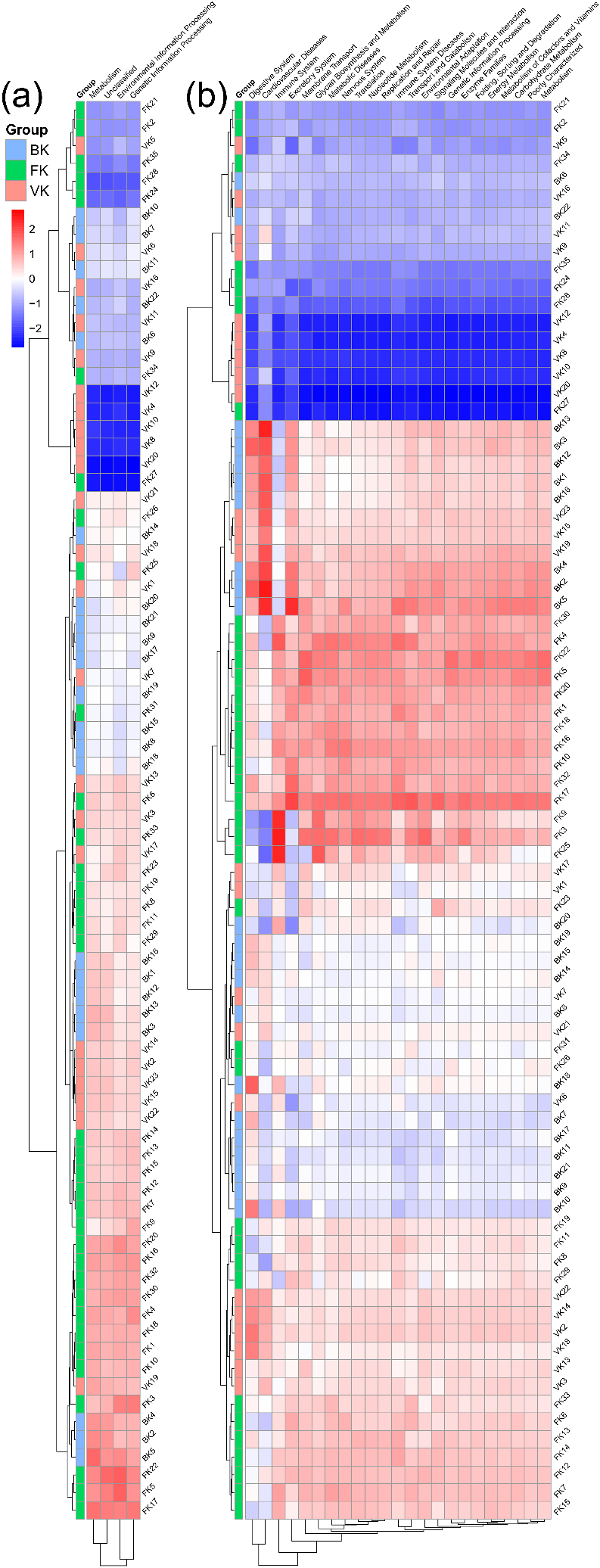


**Fig. S2. The unique composition of conjunctival bacterial gene functions can be used to distinguish microbial keratitis.** At the L1 **(a)** and L2 **(b)** levels, the heatmap shows the rank normalized abundances of the gene functions of the conjunctival bacterial microbiota related to various types of microbial keratitis. Gene functions without significant differences (determined by Kruskal–Wallis test) among the three groups were excluded. (BK: bacterial keratitis; FK: fungal keratitis; VK: viral keratitis)


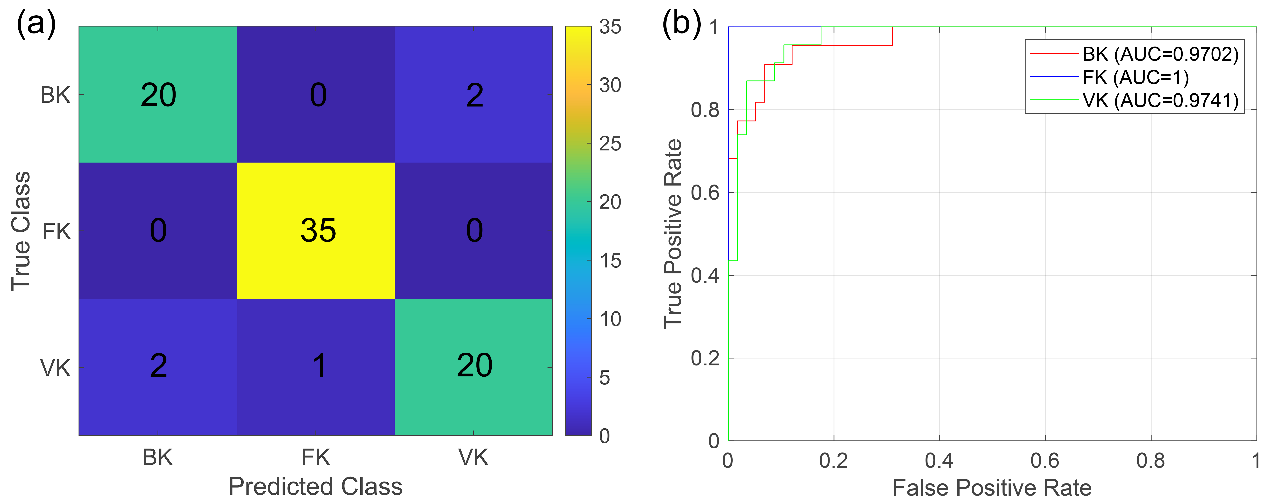


**Fig. S3 The classifier based on the relative composition of conjunctival bacterial gene functions can effectively distinguish various types of microbial keratitis.** For the confusion matrix **(a)**, a number in a grid means how many actual cases from the ordinate are judged as the abscissa. For the ROC curve **(b)**, the AUC reflects the performance of the established classifier for distinguishing various types of microbial keratitis. (CG: control group - healthy eyes; BK: bacterial keratitis; FK: fungal keratitis; VK: viral keratitis; AUC: area under the ROC curve).

## Supplementary Tables

Table S1. Detailed information of subjects

| Number | Group | Gender | Age | IVCM | Smear | Culture | Pathological examination | PCR | Clinical diagnosis |
| --- | --- | --- | --- | --- | --- | --- | --- | --- | --- |
| 1 | CG | Male | 49 | / | / | / | / | / | / |
| 2 | CG | Female | 63 | / | / | / | / | / | / |
| 3 | CG | Male | 61 | / | / | / | / | / | / |
| 4 | CG | Female | 46 | / | / | / | / | / | / |
| 5 | CG | Female | 83 | / | / | / | / | / | / |
| 6 | CG | Female | 59 | / | / | / | / | / | / |
| 7 | CG | Female | 50 | / | / | / | / | / | / |
| 8 | CG | Female | 36 | / | / | / | / | / | / |
| 9 | CG | Female | 70 | / | / | / | / | / | / |
| 10 | CG | Male | 40 | / | / | / | / | / | / |
| 11 | CG | Male | 64 | / | / | / | / | / | / |
| 12 | CG | Male | 73 | / | / | / | / | / | / |
| 13 | CG | Male | 62 | / | / | / | / | / | / |
| 14 | CG | Male | 80 | / | / | / | / | / | / |
| 15 | CG | Female | 67 | / | / | / | / | / | / |
| 16 | CG | Male | 64 | / | / | / | / | / | / |
| 17 | CG | Male | 50 | / | / | / | / | / | / |
| 18 | CG | Female | 67 | / | / | / | / | / | / |
| 19 | CG | Male | 76 | / | / | / | / | / | / |
| 20 | CG | Male | 62 | / | / | / | / | / | / |
| 21 | CG | Female | 62 | / | / | / | / | / | / |
| 22 | CG | Male | 72 | / | / | / | / | / | / |
| 23 | CG | Male | 59 | / | / | / | / | / | / |
| 24 | CG | Male | 44 | / | / | / | / | / | / |
| 25 | CG | Female | 53 | / | / | / | / | / | / |
| 26 | CG | Male | 50 | / | / | / | / | / | / |
| 27 | CG | Male | 62 | / | / | / | / | / | / |
| 28 | CG | Male | 67 | / | / | / | / | / | / |
| 29 | CG | Male | 70 | / | / | / | / | / | / |
| 30 | CG | Male | 77 | / | / | / | / | / | / |
| 31 | CG | Male | 61 | / | / | / | / | / | / |
| 32 | CG | Male | 55 | / | / | / | / | / | / |
| 33 | CG | Male | 62 | / | / | / | / | / | / |
| 34 | CG | Male | 71 | / | / | / | / | / | / |
| 35 | CG | Male | 84 | / | / | / | / | / | / |
| 36 | CG | Female | 61 | / | / | / | / | / | / |
| 37 | CG | Female | 69 | / | / | / | / | / | / |
| 38 | CG | Male | 80 | / | / | / | / | / | / |
| 39 | CG | Male | 52 | / | / | / | / | / | / |
| 40 | CG | Female | 65 | / | / | / | / | / | / |
| 41 | CG | Female | 38 | / | / | / | / | / | / |
| 42 | CG | Male | 35 | / | / | / | / | / | / |
| 43 | CG | Male | 66 | / | / | / | / | / | / |
| 44 | CG | Male | 67 | / | / | / | / | / | / |
| 45 | CG | Male | 67 | / | / | / | / | / | / |
| 46 | CG | Female | 67 | / | / | / | / | / | / |
| 47 | CG | Male | 64 | / | / | / | / | / | / |
| 48 | CG | Female | 68 | / | / | / | / | / | / |
| 49 | CG | Male | 62 | / | / | / | / | / | / |
| 50 | CG | Male | 74 | / | / | / | / | / | / |
| 51 | CG | Male | 68 | / | / | / | / | / | / |
| 52 | CG | Male | 67 | / | / | / | / | / | / |
| 53 | CG | Male | 59 | / | / | / | / | / | / |
| 54 | CG | Female | 59 | / | / | / | / | / | / |
| 55 | CG | Male | 60 | / | / | / | / | / | / |
| 56 | CG | Male | 46 | / | / | / | / | / | / |
| 57 | CG | Female | 79 | / | / | / | / | / | / |
| 58 | CG | Male | 50 | / | / | / | / | / | / |
| 59 | CG | Male | 65 | / | / | / | / | / | / |
| 60 | CG | Male | 51 | / | / | / | / | / | / |
| 61 | CG | Male | 45 | / | / | / | / | / | / |
| 62 | CG | Male | 55 | / | / | / | / | / | / |
| 63 | CG | Male | 67 | / | / | / | / | / | / |
| 64 | CG | Male | 62 | / | / | / | / | / | / |
| 65 | CG | Female | 58 | / | / | / | / | / | / |
| 66 | CG | Male | 58 | / | / | / | / | / | / |
| 67 | CG | Male | 70 | / | / | / | / | / | / |
| 68 | CG | Male | 68 | / | / | / | / | / | / |
| 69 | CG | Female | 57 | / | / | / | / | / | / |
| 70 | BK | Female | 67 | - | - | - | + | / | / |
| 71 | BK | Male | 67 | - | - | - | + | / | / |
| 72 | BK | Male | 67 | - | + | - | / | / | / |
| 73 | BK | Male | 66 | - | + | - | / | / | / |
| 74 | BK | Male | 35 | - | - | Proteus mirabilis | / | / | / |
| 75 | BK | Female | 38 | - | - | - | + | / | / |
| 76 | BK | Female | 65 | - | - | - | + | / | / |
| 77 | BK | Male | 52 | - | - | Streptococcus pneumoniae | / | / | / |
| 78 | BK | Male | 80 | - | + | - | / | / | / |
| 79 | BK | Female | 69 | - | + | - | + | / | / |
| 80 | BK | Female | 61 | - | - | - | / | / | / |
| 81 | BK | Female | 61 | - | + | - | / | / | / |
| 82 | BK | Male | 67 | - | + | - | / | / | / |
| 83 | BK | Male | 80 | - | - | Pseudomonas | / | / | / |
| 84 | BK | Female | 69 | - | - | - | + | / | / |
| 85 | BK | Male | 52 | - | - | - | + | / | / |
| 86 | BK | Female | 65 | - | - | Pseudomonas | / | / | / |
| 87 | BK | Male | 35 | - | - | - | + | / | / |
| 88 | BK | Male | 66 | - | + | - | + | / | / |
| 89 | BK | Female | 38 | - | - | Pseudomonas | / | / | / |
| 90 | BK | Male | 67 | - | - | - | + | / | / |
| 91 | BK | Female | 67 | - | - | Pseudomonas | / | / | / |
| 92 | FK | Male | 84 | + | - | Acremonium | / | / | / |
| 93 | FK | Male | 71 | + | + | Alternaria | / | / | / |
| 94 | FK | Male | 62 | + | + | - | / | / | / |
| 95 | FK | Male | 55 | + | + | Fusarium | / | / | / |
| 96 | FK | Male | 61 | + | + | Saccharomyces | / | / | / |
| 97 | FK | Male | 77 | + | + | Aspergillus | / | / | / |
| 98 | FK | Male | 70 | + | + | Fusarium | / | / | / |
| 99 | FK | Male | 67 | + | + | Fusarium | / | / | / |
| 100 | FK | Male | 62 | - | - | Fusarium | / | / | / |
| 101 | FK | Male | 50 | + | - | Fusarium | / | / | / |
| 102 | FK | Female | 53 | + | + | Fusarium | / | / | / |
| 103 | FK | Male | 44 | + | + | Alternaria | / | / | / |
| 104 | FK | Male | 59 | - | + | - | / | / | / |
| 105 | FK | Male | 72 | - | + | Alternaria | / | / | / |
| 106 | FK | Female | 62 | + | + | Alternaria | / | / | / |
| 107 | FK | Male | 62 | - | + | - | / | / | / |
| 108 | FK | Male | 76 | + | - | - | / | / | / |
| 109 | FK | Female | 67 | + | + | - | / | / | / |
| 110 | FK | Male | 50 | + | + | Alternaria | / | / | / |
| 111 | FK | Male | 64 | + | - | - | / | / | / |
| 112 | FK | Female | 67 | - | + | - | / | / | / |
| 113 | FK | Male | 80 | - | + | Alternaria | / | / | / |
| 114 | FK | Male | 62 | + | - | - | / | / | / |
| 115 | FK | Male | 73 | + | - | Fusarium | / | / | / |
| 116 | FK | Male | 64 | + | + | Fusarium | / | / | / |
| 117 | FK | Male | 40 | + | + | Fusarium | / | / | / |
| 118 | FK | Female | 70 | + | + | Alternaria | / | / | / |
| 119 | FK | Female | 36 | + | + | Alternaria | / | / | / |
| 120 | FK | Female | 50 | - | + | Alternaria | / | / | / |
| 121 | FK | Female | 59 | + | + | Alternaria | / | / | / |
| 122 | FK | Female | 83 | + | + | - | / | / | / |
| 123 | FK | Female | 46 | + | + | Alternaria | / | / | / |
| 124 | FK | Male | 61 | - | + | Aspergillus | / | / | / |
| 125 | FK | Female | 63 | + | + | Alternaria | / | / | / |
| 126 | FK | Male | 49 | + | - | - | / | / | / |
| 127 | VK | Female | 57 | - | - | - | / | / | HSV |
| 128 | VK | Male | 68 | - | - | - | / | / | HSV |
| 129 | VK | Male | 70 | - | - | Fusarium | / | HSV | HSV |
| 130 | VK | Male | 58 | - | - | - | / | HSV | / |
| 131 | VK | Female | 58 | - | - | - | / | / | HSV |
| 132 | VK | Male | 62 | - | - | - | / | HSV | / |
| 133 | VK | Male | 67 | - | - | Corynebacterium | / | HSV | HSV |
| 134 | VK | Male | 55 | - | - | - | / | / | HSV |
| 135 | VK | Male | 45 | - | - | - | / | / | HZV |
| 136 | VK | Male | 51 | - | - | - | / | HZV | HSV |
| 137 | VK | Male | 65 | - | - | - | / | / | HSV |
| 138 | VK | Male | 50 | - | - | - | / | / | HZV |
| 139 | VK | Female | 79 | - | - | - | / | HSV | / |
| 140 | VK | Male | 46 | - | - | - | / | / | HSV |
| 141 | VK | Male | 60 | - | - | - | / | / | HZV |
| 142 | VK | Female | 59 | - | - | - | / | HSV | / |
| 143 | VK | Male | 59 | - | - | - | / | HSV | / |
| 144 | VK | Male | 67 | - | - | - | / | / | HSV |
| 145 | VK | Male | 68 | - | - | - | / | / | HZV |
| 146 | VK | Male | 74 | - | - | - | / | / | HSV |
| 147 | VK | Male | 62 | - | - | - | / | / | HSV |
| 148 | VK | Female | 68 | - | - | - | / | / | HZV |
| 149 | VK | Male | 64 | - | - | - | / | HZV | HSV |
| IVCM: *in vivo* confocal microscopy; CG: control group; BK: bacterial keratitis; FK: fungal keratitis; VK: viral keratitis BK: HSV: herpes simplex virus; HZV: herpes zoster virus; (+): positive result; (-) negative result; (/): not examined | | | | | | | | | |
| Clinical diagnosis refers to the diagnosis according to medical history and constitutional symptoms | | | | | | | | | |

**Table S2. Detailed information of software parameters**

| Software | Version | Parameters |
| --- | --- | --- |
| Trimmomatic | 0.35 | TRAILING:3 SLIDINGWINDOW:50:20 MINLEN:50 |
| Flash | 1.2.11 | -M 200 |
| QIIME(multiple rarefactions.py) | 1.8.0 | -m 10 -x 1000 -n 10 |
| QIIME(split libraries) | 1.8.0 | -H 8 -l 200 |
| Vsearch | 2.4.2 | --id 0.97 |
| pynast | 0.1 | - |

**Table S3. Weighted NSTI values of each sample.**

| CG samples | Weighted NSTI | BK samples | Weighted NSTI | FK samples | Weighted NSTI | VK samples | Weighted NSTI |
| --- | --- | --- | --- | --- | --- | --- | --- |
| CG1 | 0.1427375 | BK1 | 0.1431329 | FK1 | 0.1402567 | VK1 | 0.1413583 |
| CG2 | 0.1259858 | BK10 | 0.0979294 | FK10 | 0.1519068 | VK2 | 0.1168388 |
| CG3 | 0.1240764 | BK11 | 0.125675 | FK11 | 0.1321015 | VK3 | 0.1334043 |
| CG4 | 0.1326903 | BK12 | 0.142161 | FK12 | 0.1453619 | VK4 | 0.1069171 |
| CG5 | 0.1344889 | BK13 | 0.1504548 | FK13 | 0.1268438 | VK5 | 0.1437204 |
| CG6 | 0.1449145 | BK14 | 0.1276523 | FK14 | 0.1391888 | VK6 | 0.0840083 |
| CG7 | 0.1205402 | BK15 | 0.130337 | FK15 | 0.1548337 | VK7 | 0.1543923 |
| CG8 | 0.1341474 | BK16 | 0.1334831 | FK16 | 0.1368339 | VK8 | 0.1208447 |
| CG9 | 0.1425677 | BK17 | 0.111127 | FK17 | 0.1442889 | VK9 | 0.1342612 |
| CG10 | 0.1333036 | BK18 | 0.1066992 | FK18 | 0.1364204 | VK10 | 0.1194208 |
| CG11 | 0.140523 | BK19 | 0.1307259 | FK19 | 0.1416244 | VK11 | 0.1394089 |
| CG12 | 0.1504039 | BK2 | 0.144303 | FK2 | 0.1341625 | VK12 | 0.140914 |
| CG13 | 0.1422169 | BK20 | 0.1534594 | FK20 | 0.1286977 | VK13 | 0.1462163 |
| CG14 | 0.1436476 | BK21 | 0.1175634 | FK21 | 0.1217659 | VK14 | 0.1165474 |
| CG15 | 0.1439091 | BK22 | 0.1215817 | FK22 | 0.1189056 | VK15 | 0.1368783 |
| CG16 | 0.1312031 | BK3 | 0.1517068 | FK23 | 0.0948577 | VK16 | 0.1296679 |
| CG17 | 0.1207313 | BK4 | 0.1453711 | FK24 | 0.1166779 | VK17 | 0.1532127 |
| CG18 | 0.1480643 | BK5 | 0.14871 | FK25 | 0.1565648 | VK18 | 0.1219217 |
| CG19 | 0.1508266 | BK6 | 0.1195659 | FK26 | 0.0983295 | VK19 | 0.1309908 |
| CG20 | 0.1492924 | BK7 | 0.1191631 | FK27 | 0.122904 | VK20 | 0.1168451 |
| CG21 | 0.1356522 | BK8 | 0.1409548 | FK28 | 0.1141193 | VK21 | 0.1432877 |
| CG22 | 0.1135732 | BK9 | 0.1132319 | FK29 | 0.1240518 | VK22 | 0.119258 |
| CG23 | 0.0792031 |  |  | FK3 | 0.1986802 | VK23 | 0.1414421 |
| CG24 | 0.1153418 |  |  | FK30 | 0.1261894 |  |  |
| CG25 | 0.0886574 |  |  | FK31 | 0.153688 |  |  |
| CG26 | 0.122214 |  |  | FK32 | 0.1121099 |  |  |
| CG27 | 0.1210798 |  |  | FK33 | 0.1566875 |  |  |
| CG28 | 0.1332247 |  |  | FK34 | 0.1176259 |  |  |
| CG29 | 0.1093017 |  |  | FK35 | 0.1152495 |  |  |
| CG30 | 0.118649 |  |  | FK4 | 0.1467805 |  |  |
| CG31 | 0.1218032 |  |  | FK5 | 0.116501 |  |  |
| CG32 | 0.0991376 |  |  | FK6 | 0.1552018 |  |  |
| CG33 | 0.1162687 |  |  | FK7 | 0.1486112 |  |  |
| CG34 | 0.116703 |  |  | FK8 | 0.1378694 |  |  |
| CG35 | 0.118961 |  |  | FK9 | 0.1647725 |  |  |
| CG36 | 0.1245786 |  |  |  |  |  |  |
| CG37 | 0.144884 |  |  |  |  |  |  |
| CG38 | 0.1467233 |  |  |  |  |  |  |
| CG39 | 0.1294448 |  |  |  |  |  |  |
| CG40 | 0.1309808 |  |  |  |  |  |  |
| CG41 | 0.1267346 |  |  |  |  |  |  |
| CG42 | 0.1483505 |  |  |  |  |  |  |
| CG43 | 0.111334 |  |  |  |  |  |  |
| CG44 | 0.1213194 |  |  |  |  |  |  |
| CG45 | 0.1162561 |  |  |  |  |  |  |
| CG46 | 0.1394986 |  |  |  |  |  |  |
| CG47 | 0.1179234 |  |  |  |  |  |  |
| CG48 | 0.1255514 |  |  |  |  |  |  |
| CG49 | 0.1211783 |  |  |  |  |  |  |
| CG50 | 0.1335348 |  |  |  |  |  |  |
| CG51 | 0.1210158 |  |  |  |  |  |  |
| CG52 | 0.1273758 |  |  |  |  |  |  |
| CG53 | 0.1237882 |  |  |  |  |  |  |
| CG54 | 0.1278505 |  |  |  |  |  |  |
| CG55 | 0.1256561 |  |  |  |  |  |  |
| CG56 | 0.1113878 |  |  |  |  |  |  |
| CG57 | 0.1315244 |  |  |  |  |  |  |
| CG58 | 0.1310701 |  |  |  |  |  |  |
| CG59 | 0.1287231 |  |  |  |  |  |  |
| CG60 | 0.1345694 |  |  |  |  |  |  |
| CG61 | 0.1284893 |  |  |  |  |  |  |
| CG62 | 0.1333616 |  |  |  |  |  |  |
| CG63 | 0.1370778 |  |  |  |  |  |  |
| CG64 | 0.1129176 |  |  |  |  |  |  |
| CG65 | 0.1131657 |  |  |  |  |  |  |
| CG66 | 0.1332041 |  |  |  |  |  |  |
| CG67 | 0.1305721 |  |  |  |  |  |  |
| CG68 | 0.130488 |  |  |  |  |  |  |
| CG69 | 0.1004549 |  |  |  |  |  |  |
